# Knowledge of and attitude to foot care amongst Type 2 diabetes patients attending a university-based primary care clinic in Nigeria

**DOI:** 10.4102/phcfm.v2i1.175

**Published:** 2010-10-29

**Authors:** Rabi I. Ekore, Ikeoluwapo O. Ajayi, Ayo Arije, John O. Ekore

**Affiliations:** 1 University Health Service, Jaja Clinic, University Of Ibadan, Nigeria; 2 Dept of Epidemiology, Medical Statistics and Environmental Health, University of Ibadan, Nigeria; 3 Department of Medicine, University of Ibadan, Nigeria; 4 Dept. of Psychology, University of Ibadan, Nigeria

**Keywords:** attitude, diabetic foot care, education, knowledge, Type 2 diabetes mellitus

## Abstract

**Background:**

Individuals living with diabetes mellitus are at an increased risk of developing foot ulcers and cardiovascular complications or a neuropathy that may result in amputations. These complications have been shown to be already present in about 10% of diabetic patients at the time of diagnosis.

**Objectives:**

This study was carried out to determine the level of awareness and attitude to foot care among adult diabetic patients attending a university health centre (i.e. a primary care centre) and to emphasise the ever-present need for health education and promotion and early complication detection (especially of foot problems) among diabetic patients.

**Method:**

A descriptive cross-sectional, clinic-based study was carried out at the University of Ibadan Health Centre (Jaja Clinic). The study population consisted of consenting adult diabetic patients. Data were collected by the self-administration of structured questionnaires to eligible subjects and were analysed using the SPSS v.15software. Appropriate statistics were employed to analyse the collected data.

**Results:**

A total of 137 patients participated in the study and ranged in age from 37 to 75 years, with the mean ± SD age being 58.2 ± 9.2 years. Of the participants, 98 (71.5%) were men and 39 (28.5%) were women; all of the participants were married. The duration of illness ranged from 1 year to 20 years, with the median duration of illness being 3 ± 1.7 years. One hundred and twenty-six (92%) patients had never received any education on foot care from their healthcare providers, while 11 (8%) had received some form of foot care education. Among those who had never received any foot care education, 92 (73%) had been diabetic for 1–5 years, while the remaining 34 (27%) had been diabetic for 6 – 20 years. Of the foot care measures that were known, 35 (25.5%) patients knew to wash their feet daily and dry in between the toes thoroughly, 31 (22.6%) knew not to go outdoors barefooted, 27 (19.7%) checked their feet daily, 27 (19.7%) checked inside their shoes daily, 8 (5.8%) consciously made an effort to avoid injuries to their feet and 4 (2.9%) clipped their toenails with care.

**Conclusion:**

The results of this study showed that awareness of foot care measures is very poor among known diabetic patients and this is largely due to a lack of education of the patients by their health care providers.

## INTRODUCTION

Individuals living with diabetes mellitus are at an increased risk of developing foot ulcers and cardiovascular complications or a neuropathy that may result in amputations.^1^ These complications have been shown to be already present in about 10% of diabetic patients at the time of diagnosis.^2^ The United Kingdom prospective diabetes study (UKPDS) revealed that about 20% of diabetics suffer from diabetic neuropathy in the first 4 years after diagnosis, which increased to about 50% or more after 15 years.^3^

A study by Lavery et al.^4^ on the incidence of foot pathology in Mexican–Americans and non-Hispanic Whites revealed an incidence of 5.9 foot amputations per 1000 persons with diabetes per year. In view of ‘the menace of diabetic gangrene’, Joslin^5^ vehemently advocated intensive education of patients on the cleanliness and care of their feet in order to minimise the occurrence of diabetic gangrene. Similarly, among the recommendations of ‘The St Vincent declaration’ in 1989 were the reduction by one half in the rate of limb amputations for diabetic gangrene and the implementation of effective measures for the prevention of costly complications of diabetes mellitus.^6^

Studies^2, 7^ have revealed a complete lack of information about preventive or early treatment measures in most of the diabetic patients who have had their lower-leg amputated. In a study to assess the quality of care provided to diabetic patients by family physicians at the University of Lebanon Health Clinic, physicians were noted to have documented patient instructions on foot care, diet, exercise and diabetes self-care poorly, implying that they may not have given these instructions to the patients in the first instance.^7^ This underscores the need to determine the level of awareness of diabetic patients about the prevention of foot ulcers in order to plan an intervention strategy. Educating patients about proper foot care and periodic self-foot examinations has been found to be an effective method of intervention that can prevent (foot) ulceration.^8^ This activity falls under the domain of preventive health care services expected of a University Health Service.

## METHOD

This paper reports a descriptive, clinic-based cross-sectional study that took place at the University of Ibadan, Health Centre's Jaja Clinic. It is a primary care centre that caters for students and staff of the university, as well as members of the surrounding communities. The clinic employs 8 full-time medical doctors (including 3 family physicians), 4 locum doctors, 20 nurses, 2 pharmacists, 4 pharmacy technicians, 6 medical records officers and 6 hospital assistants. The clinic comprises the following units: outpatients, the pharmacy, nursing, public health, the laboratory, environmental health, the administrative and medical records unit. Services provided by the clinic include the diagnosis and treatment of medical and minor surgical ailments, especially common ailments, such as malaria, respiratory tract infections, chronic non-communicable diseases, such as hypertension, diabetes mellitus and bronchial asthma, the immunisation of infants and adults, fumigation services, and the evacuation of filled sewage pits. The Damien Foundation also operates a centre within the clinic for the treatment of patients with tuberculosis. Patients are usually examined in consultation rooms, with each patient seeing the first available physician. There are no disease-specific clinics or clinic days and no pre-consultation health talks. All patients are taken directly from the medical records unit to the consultation rooms, each to await their turn for consultation. Diabetic patients usually have their fasting blood glucose checked in the laboratory prior to consultation. Apart from blood glucose, other laboratory tests that may be conducted include full blood count, total serum cholesterol, serum electrolyte and urea and urinalysis. After consultation with a physician, each patient is given an appointment, the length of which depends on the current value of the fasting blood glucose.

The respondents included consenting Type 2 diabetes mellitus adult patients being managed at the health centre; newly diagnosed Type 2 diabetic patients were excluded from the study. Data were collected by self-administration of questionnaires to assess the patients’ level of awareness of foot care measures, whether they have ever received foot care education at the clinic and their attitudes towards practising foot care measures. Data were analysed using the SPPS v.15. Frequency distribution of the variables was performed to describe the data and cross-tabulation was conducted to compare variables.

## RESULTS

A total of 137 patients participated in the study and ranged in age from 37 to 75 years, with the mean ± SD age being 58.2 ± 9.2 years. Of the participants, 98 (71.5%) were men and 39 (28.5%) were women; all of the participants were married. The duration of illness ranged from 1 to 20 years, with the median duration of illness being 3 ± 1.7 years. Seventy patients (51.1%) were being managed on oral hypoglycaemic agents alone, while 55 (40.1%) were receiving a combination of diet and oral hypoglycaemic agents and 8 (5.8%) were being managed on diet alone. One hundred and twenty-six (92%) patients mentioned they had never received any education on foot care from their health care providers, while 11 (8%) had received some form of foot care education. Among those who had never received any foot care education, 92 (73%) had been diabetic for 1–5 years, while the remaining 34 (27%) had been diabetic for 6–20 years. Of the foot care measures that were known, 35 (25.5%) patients knew to wash their feet daily and dry in between the toes thoroughly, 31 (22.6%) knew not to go outdoors barefooted, 27 (19.7%) checked their feet daily, 27 (19.7%) checked inside their shoes daily, 8 (5.8%) consciously made an effort to avoid injuries to their feet and 4 (2.9%) clipped their toenails with care.

Table 1 depicts the age range of the patients in relation to the percentage awareness of foot care measures, while Table 2 shows the duration of diagnosis in relation to the level of awareness of the patients.

**TABLE 1:** Age group of patients with some awareness of foot care measures

Age (years)	Avoid walking barefoot		Wash feet daily		Check feet daily		Check shoes daily		Avoid injuries		Clip toenails	
					
	*n*	*%*	*n*	*%*	*n*	*%*	*n*	*%*	*n*	*%*	*n*	*%*
≤ 45	4	12.9	8	22.9	4	14.8	8	29.6	0	0	0	0
46–55	4	12.9	4	11.4	8	29.6	4	14.8	4	50	4	100
56–65	19	61.3	19	54.3	8	29.6	7	25.9	0	0	0	0
66–75	4	12.9	4	11.4	7	25.9	8	29.6	4	50	0	0

**Total**	**31**	**100**	**35**	**100**	**27**	**100**	**27**	**100**	**8**	**100**	**4**	**100**

Values are given as means (*n* = 00.00)

Percentages are given as means (% = 00.00)

**TABLE 2:** Duration of diabetes and awareness of foot care measures

Duration (years)	Avoid walking barefoot		Wash feet daily		Check feet daily		Check shoes daily		Avoid injuries		Clip toenails	
					
	*n*	*%*	*n*	*%*	*n*	*%*	*n*	*%*	*n*	*%*	*n*	*%*
1–5	23.00	74.20	31	88.6	15	55.6	19	70.4	4	50		0
6–10	4.00	12.90	4	11.4	4	14.8	4	14.8	0	0	4	0
11–15	4.00	12.90	0	0.0	4	14.8	4	14.8	4	5	0	0
16–20	0.00	0.00	0	0.0	4	14.8	0	0.0	0	0	0	0

**Total**	**31.00**	**100.00**	**35**	**100**	**27**	**100**	**27**	**100**	**8**	**100**	**4**	**100**

Values are given as means (*n* = 00.00)

Percentages are given as means (% = 00.00)

## DISCUSSION

Daily foot care is essential for preventing complications of diabetic neuropathy and vascular insufficiency.^9^ This study revealed that as many as 92% of the diabetic patients receiving care at the study site had never received any form of education about foot care from their health care providers. This is much higher than the result obtained from a study conducted among Indian diabetics, which revealed that about 44.7% of patients had not received previous foot care education.^10^ Considering the high prevalence of morbidity and mortality resulting from foot complications among diabetic patients, this finding is alarming. Factors responsible for this situation may include the fact that,(1) there are no organised health education sessions prior to consultation, (2) there are no disease-specific clinic days, (3) there is no continuous care for a particular patient by the same physician and (4) a total lack of interest in health education by the physicians for one reason or the other. A number of the patients who had some knowledge of foot care procedures had obtained this information from the Internet or books. This significant lack of education on foot care is unacceptable, especially considering the fact that diabetic foot syndrome not only poses serious medical problems, but also has a major socio-economic impact, by virtue of the number of hospital visits and admissions and restriction of mobility with its attendant effect on psychological well-being and quality of life.^2^ The study done at the University of Lebanon Health Clinic concluded that there is an urgent need for interventions to improve management and documentation in diabetes care in order to achieve early detection and prevention of complications.^7^

### Recommendations

Health care teams of institutional health care services, especially at a university level, should endeavour to incorporate foot care education for diabetic patients into their daily practice, in order to prevent or reduce the occurrence of diabetic foot syndrome.

## CONCLUSION

In this study, awareness of foot care measures is very poor among known diabetic patients and this is largely due to a lack of education of the patients by their health care providers. Health care workers particularly physicians should endeavour to give their diabetic patients necessary health education about foot care in order to reduce the burden of foot complications among diabetic patients. If too busy to give health education, they should at least ensure some other health worker does it.
